# Description and phylogeny of three new species of *Synophis* (Colubridae, Dipsadinae) from the tropical Andes in Ecuador and Peru

**DOI:** 10.3897/zookeys.546.6533

**Published:** 2015-12-16

**Authors:** Omar Torres-Carvajal, Lourdes Y. Echevarría, Pablo J. Venegas, Germán Chávez, Jeffrey D. Camper

**Affiliations:** 1 Museo de Zoología, Escuela de Ciencias Biológicas, Pontificia Universidad Católica del Ecuador, Avenida 12 de Octubre y Roca, Apartado 17-01-2184, Quito-Ecuador; 2 División de Herpetología-Centro de Ornitología y Biodiversidad (CORBIDI), Santa Rita N˚105 Of. 202, Urb. Huertos de San Antonio, Surco, Lima-Perú; 3 Department of Biology, Francis Marion University, Florence, South Carolina 29506-USA

**Keywords:** Andes, Dipsadinae, Ecuador, new species, Peru, snakes, *Synophis*, systematics

## Abstract

The discovery of three new species of *Synophis* snakes from the eastern slopes of the tropical Andes in Ecuador and Peru is reported. All previous records of *Synophis bicolor* from eastern Ecuador correspond to *Synophis bogerti* **sp. n.**, which occurs between 1000–1750 m along a large part of the Amazonian slopes of the Ecuadorian Andes. In contrast, *Synophis zamora* **sp. n.** is restricted to southeastern Ecuador, including Cordillera del Cóndor, between 1543–1843 m. *Synophis insulomontanus* **sp. n.** is from the eastern slopes of the Andes in central and northern Peru, between 1122–1798 m, and represents the first record of *Synophis* from this country. All three new species share in common a large lateral spine at the base of the hemipenial body. A molecular phylogenetic tree based on three mitochondrial genes is presented, including samples of *Diaphorolepis wagneri*. Our tree strongly supports *Synophis* and *Diaphorolepis* as sister taxa, as well as monophyly of the three new species described here and *Synophis calamitus*. Inclusion of *Synophis* and *Diaphorolepis* within Dipsadinae as sister to a clade containing *Imantodes*, *Dipsas*, *Ninia*, *Hypsiglena* and *Pseudoleptodeira* is also supported.

## Abstract (es)

Se reporta el descubrimiento de tres especies nuevas de serpientes *Synophis* de las estribaciones orientales de los Andes tropicales en Ecuador y Perú. Todos los registros previos de *Synophis bogerti* del oriente ecuatoriano corresponden a *Synophis bogerti* **sp. n.**, la cual ocurre entre 1000–1750 m a lo largo de gran parte de las estribaciones amazónicas de los Andes ecuatorianos. En contraste, *Synophis zamora* **sp. n.** se restringe al suroriente de Ecuador, incluyendo la Cordillera del Cóndor, entre 1543–1843 m. *Synophis insulomontanus* **sp. n.** es de las estribaciones orientales de los Andes del centro y norte del Perú, entre 1122–1798 m, y representa el primer registro de *Synophis* para este país. Todas las tres especies nuevas comparten en común una espina lateral larga en la base del cuerpo del hemipene. Un árbol molecular filogenético, basado en tres genes mitocondriales es presentado, incluyendo muestras de *Diaphorolepis wagneri*. Nuestro árbol apoya fuertemente a *Synophis* y *Diaphorolepis* como taxa hermanos, así como la monofilia de las tres especies descritas y de *Synophis calamitus*. La inclusión de *Synophis* y *Diaphorolepis* dentro de Dipsadinae, como hermanas a un clado que contiene a *Imantodes*, *Dipsas*, *Ninia*, *Hypsiglena* y *Pseudoleptodeira* también es apoyada.

## Introduction

With only four recognized species, *Synophis* is among the least speciose snake groups formally recognized as genera in South America. Species of *Synophis* are known to occur in the Andes of Colombia and Ecuador between approximately 460–2200 m (Hillis 1990). Whereas *Synophis plectovertebralis* and *Synophis calamitus* are endemic to Colombia and Ecuador, respectively (Hillis 1990; Sheil and Grant 2001), *Synophis bicolor* and *Synophis lasallei* have been reported in both countries (Bogert 1964; Nicéforo-María 1970).

The taxonomic identity of specimens currently assigned to *Synophis bicolor* (Peracca 1896) has been problematic for two reasons. First, the type locality of this species is ambiguous (‘America meridionale’) preventing the collection of topotypes for comparison. Second, there is significant morphological variation between specimens of *Synophis bicolor* from Colombia and Ecuador. In his taxonomic review of *Synophis* and *Diaphorolepis* Bogert (1964) noted some differences between specimens from Ecuador and the holotype of *Synophis bicolor* (in parentheses): 10–11 infralabials (9), 160–166 ventrals (180), 100–118 subcaudals (136), 24–27 maxillary teeth (16), 14 palatine teeth (9-10), 32–34 pterygoid teeth (21–22). Based on this variation, Bogert (1964) recognized that “specimens tentatively referred to *Synophis bicolor* might not be conspecific”. Subsequently, Niceforo-María (1970) reported the first specimen of *Synophis bicolor* from Colombia and noted that the numbers of ventrals and subcaudals (184 and 127, respectively) are more similar to the holotype than the specimens from Ecuador. This suggests that at least some populations from Ecuador currently assigned to *Synophis bicolor* represent one or more similar undescribed species instead.

The study of *Synophis* has been hampered by the paucity of specimens in collections, possibly because of low densities or semifossorial habits (Sheil and Grant 2001). Recent collections in poorly explored areas of the Amazonian slopes of the Andes from Ecuador and Peru yielded a few specimens of *Synophis* that are similar in morphology to specimens of *Synophis bicolor* previously reported from eastern Ecuador (Bogert 1964). Based on these recent collections, including the first specimens of *Synophis* from Peru, we combine evidence from morphology and phylogenetic analyses of DNA sequence data to describe three new species of *Synophis*.

## Materials and methods

### Morphological data

All type specimens of the new species are deposited at Museo de Zoología, Pontificia Universidad Católica del Ecuador, Quito (QCAZ); and Centro de Ornitología y Biodiversidad (CORBIDI), Lima, Peru. Other specimens used for comparisons are listed in Appendix I. Sex was determined by observation of hemipenes from X-ray images or by noting the presence of everted hemipenes. Snout-vent length (SVL) and tail length were measured with a ruler and recorded to the nearest millimeter. Other measurements were taken with digital calipers (±0.01 mm). We prepared partially everted hemipenes following Zaher and Prudente (2003), and immersed them for 6 h in an alcoholic solution of Alizarin Red to dye the calcareous ornaments (e.g., spines). Terminology for hemipenis description follows Dowling and Savage (1960), as augmented by Zaher (1999). Data on the hemipenes of *Synophis calamitus* and *Synophis lasallei* were taken from the literature (Zaher 1999).

### Molecular data

Total genomic DNA was digested and extracted from liver or muscle tissue using a guanidinium isothiocyanate extraction protocol. Tissue samples were first mixed with Proteinase K and lysis buffer and digested overnight prior to extraction. DNA samples were quantified using a Nanodrop® ND-1000 (NanoDrop Technologies, Inc), re-suspended and diluted to 25 ng/ul in ddH2O prior to amplification.

We amplified 2173 nucleotides (nt) encompassing three mitochondrial genes, NADH dehydrogenase subunit 4 (*ND4*, 567 nt), cytochrome b (*cyt-b*, 1069 nt) and the ribosomal large subunit (*16S*, 537 nt) from 10 individuals of the three new species described in this paper, five individuals of *Synophis calamitus*, and two of *Diaphorolepis wagneri*. Cyt-b was amplified using the primers GluDG, LGL765, L14910, H16064 (Bickham et al. 1995; Burbrink et al. 2000; Palumbi 1996; Parkinson et al. 2002), and primer CytbV 5’-GGCGAATAAGGAAGTATCATT-3’ designed by A. Fouquet; ND4 was amplified using the primers ND4, LEU and ND412931L (Arévalo et al. 1994; Blair et al. 2009); and 16S was amplified with 16SF.0 and 16SR.0 (Pellegrino et al. 2001; Whiting et al. 2003). Amplification of genomic DNA consisted of an initial cycle at 94–96 °C for 3–5 min, followed by 35–40 cycles of a denaturation at 94 °C for 30–40 s, annealing at 51–52 °C for 40–60 s, and extension at 72 °C for 40–60 s, as well as a final extension at 72 °C for 7–10 min. Genbank accession numbers of sequences generated in this study are shown in Table 1.

**Table 1.** Vouchers, locality data, and GenBank accession numbers of new sequences obtained for this study.

Taxon	Voucher	Locality^a^	Genbank number			GenSeq Nomenclature
			*cyt-b*	*ND4*	*16S*	
*Diaphorolepis wagneri*	QCAZ11956	Ecuador: Imbabura: Reserva Manduriacu	KT345360	KT345377	KT345343	genseq-4
*Diaphorolepis wagneri*	QCAZ11961	Ecuador: Imbabura: Reserva Manduriacu	KT345361	KT345378	KT345344	genseq-4
*Synophis bogerti*	QCAZ5072	Ecuador: Napo: Wildsumaco Wildlife Sactuary	KT345372	KT345389	KT345355	genseq-2
*Synophis bogerti*	QCAZ12791	Ecuador: Napo: Wildsumaco Wildlife Sactuary	KT345365	KT345382	KT345348	genseq-1
*Synophis bogerti*	QCAZ13323	Ecuador: Morona Santiago: Sardinayacu, Parque Nacional Sangay	KT345368	KT345385	KT345351	genseq-2
*Synophis bogerti*	QCAZ13585	Ecuador: Pastaza: Zarentza, Parque Nacional Llanganates	KT345369	KT345386	KT345352	genseq-2
*Synophis bogerti*	QCAZ13586	Ecuador: Pastaza: Zarentza, Parque Nacional Llanganates	KT345370	KT345387	KT345353	genseq-2
*Synophis calamitus*	QCAZ3875	Ecuador: Cotopaxi: Naranjito, Bosque Integral Otonga	KT345371	KT345388	KT345354	genseq-4
*Synophis calamitus*	QCAZ5847	Ecuador: Carchi: 14 km El Chical-Gualchán	KT345373	KT345390	KT345356	genseq-4
*Synophis calamitus*	QCAZ8098	Ecuador: Pichincha: El Cedral	KT345374	KT345391	KT345357	genseq-4
*Synophis calamitus*	QCAZ10508	Ecuador: Pichincha: El Cedral	KT345362	KT345379	KT345345	genseq-4
*Synophis calamitus*	QCAZ11931	Ecuador: Pichincha: Reserva Ecológica Santa Lucía	KT345363	KT345380	KT345346	genseq-4
*Synophis insulomontanus*	CORBIDI9223	Perú: San Martin: Picota: Puesto de Control 16 Chambirillo (Cordillera Azul)	KT345366	KT345383	KT345349	genseq-2
*Synophis insulomontanus*	CORBIDI13940	Perú: Huánuco: Pachitea: Cordillera El Sira	KT345367	KT345384	KT345350	genseq-1
*Synophis zamora*	QCAZ9174	Ecuador: Zamora Chinchipe: Las Orquídeas	KT345375	KT345392	KT345358	genseq-1
*Synophis zamora*	QCAZ9175	Ecuador: Zamora Chinchipe: Las Orquídeas	KT345376	KT345393	KT345359	genseq-2
*Synophis zamora*	QCAZ12773	Ecuador: Zamora Chinchipe: Numbami reserve, 18 km Zamora-Romerillos	KT345364	KT345381	KT345347	genseq-2

a See species accounts and Appendix I for geographic coordinates and altitude data.

Additionally, we obtained from GenBank sequences of 12 Dipsadinae taxa and *Natrix natrix*, which was used to root the tree following the phylogenetic hypothesis presented by Pyron et al. (2013). We only selected those Dipsadinae species that had sequence data for all three genes included in our analyses. Gene regions of outgroup taxa included in phylogenetic analyses along with their GenBank accession numbers are shown in Table 2.

**Table 2.** Outgroup taxa used in this study along with their GenBank accession numbers.

Taxon	Genbank number		
	*cyt-b*	*ND4*	*16S*
Natricinae			
*Natrix natrix*	AY487723	AY487799	KJ128951
Dipsadinae			
*Alsophis antillensis*	FJ416726	FJ416800	FJ416702
*Contia tenuis*	AF471095	AF402656	AY577030
*Diadophis punctatus*	AF471094	AF258889	AF544793
*Dipsas catesbyi*	EF078537	EF078585	JQ598868
*Farancia abacura*	U69832	DQ902307	Z46491
*Hypsiglena chlorophaea*^a^	KJ486459	KJ486459	KJ486459
*Imantodes cenchoa*^a^	EU728586	EU728586	EU728586
*Ninia atrata*	GQ334553	GQ334659	JQ598882
*Oxyrhopus*	GQ334554	GQ334660	GU018170
*Pseudoleptodeira latifasciata*^a^	NC013981	NC013981	NC013981
*Thermophis zhaoermii*^a^	GQ166168	GQ166168	GQ166168
*Uromacer catesbyi*	FJ416714	FJ416788	AF158523

a Sequences extracted from whole mitochondrial genomes.

### Phylogenetic analyses

Data were assembled and aligned in Geneious v7.1.7 (Kearse et al. 2012) under default settings for MAFFT (Katoh and Toh 2010). *ND4* and *cyt-b* sequences were translated into amino acids for confirmation of alignment. The best-fit nucleotide substitution models and partitioning scheme were chosen simultaneously using PartitionFinder v1.1.1 (Lanfear et al. 2012) under the Bayesian Information Criterion (BIC). The “greedy” algorithm was used with branch lengths of alternative partitions “linked” to search for the best-fit scheme, which consisted of three partitions: (i) *16S*, 3^rd^ codon positions of both *cyt-b* and *ND4* [GTR + I + G]; (ii) 2^nd^ codon positions of both *cyt-b* and *ND4* [K81uf+G]; and (iii) 1^st^ codon positions of both *cyt-b* and *ND4* [HKY + I + G]. Bayesian inference was used to obtain a phylogenetic tree of the combined dataset using the program MrBayes v3.2.1 (Ronquist et al. 2012). All parameters were unlinked between partitions (except topology and branch lengths), and rate variation (prset ratepr = variable) was invoked. Four independent runs, each with four MCMC chains, were run for five million generations, sampling every 1,000 generations. Results were analyzed in Tracer to assess convergence and effective sample sizes (ESS) for all parameters. Additionally, we verified that the average standard deviation of split frequencies between chains and the potential scale reduction factor (Gelman and Rubin 1992) of all the estimated parameters approached values of ≤ 0.01 and 1, respectively. Of the 5,000 trees resulting per run, 25% were arbitrarily discarded as “burn-in”. The remaining trees were used to calculate posterior probabilities (PP) for each bipartition in a 50% majority-rule consensus tree. The phylogenetic tree was visualized and edited using FigTree v1.4.2 (Rambaut 2014).

## Results

The taxonomic conclusions of this study are based on the observation of morphological features and color patterns, as well as inferred phylogenetic relationships. We consider this information as species delimitation criteria following a general lineage or unified species concept (de Queiroz 1998; 2007).

### Synophis bogerti sp. n.

Taxon classificationAnimaliaSquamataColubridae

http://zoobank.org/05AC659D-BA2E-4953-B2EE-182ABFBF2324

Synophis bogerti Proposed standard English name: Bogert’s fishing snakes
Synophis bogerti Proposed standard Spanish name: Serpientes pescadoras de Bogert
Synophis bicolor (part)—Bogert (1964): 515.

#### Holotype.

**Figure 1.**
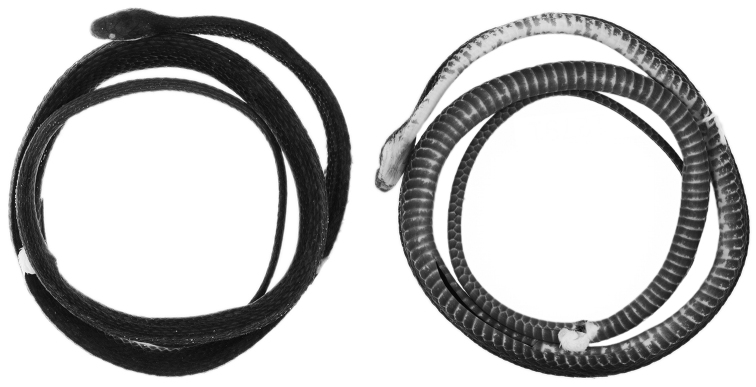
Holotype (QCAZ 12791, adult male, SVL = 367 mm) of *Synophis bogerti* sp. n. in dorsal (left) and ventral (right) views. Photographs by Omar Torres-Carvajal.

**Figure 2.**
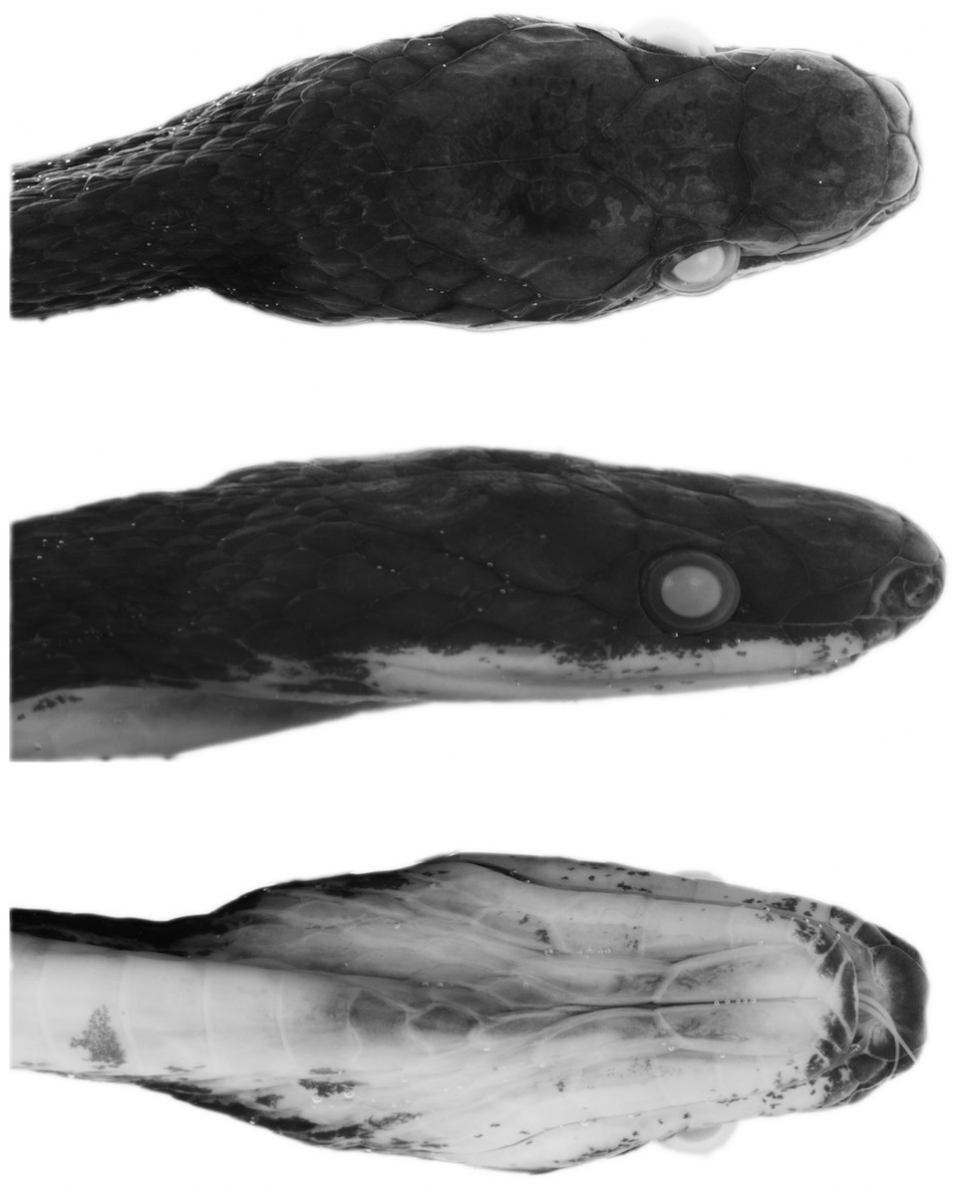
Head of holotype of *Synophis bogerti* sp. n. (QCAZ 12791) in dorsal (top), lateral (middle) and ventral (bottom) views. Photographs by Omar Torres-Carvajal.

Ecuador: Provincia Napo: QCAZ 12791 (Figs 1, 2), adult male from Wildsumaco Wildlife Sanctuary, sendero Coatí (0°38'8.40"S, 77°31'19.20"W, 1000 m), collected on 18 July 2014 by J. D. Camper.

#### Paratypes.

Ecuador: Provincia Morona Santiago: QCAZ 13323 adult male from Laguna Cormorán, Sardinayacu, Parque Nacional Sangay (2°4'17.51"S, 78°12'57.24"W, 1747 m), collected on 16 January 2015 by J. Pinto, D. Velalcázar and D. Nuñez. Provincia Napo: QCAZ 3511, adult female from Cordillera de los Guacamayos (0°37'40.16"S, 77°50'0.98"W, 1200 m), collected on 1 August 1995 by S. Burneo and M. Díaz; QCAZ 5072 adult male from Wildsumaco Wildlife Sanctuary (0°41'9.26"S, 77°35'54.93"W, 1250 m), collected on 26 July 2012 by J. D. Camper; QCAZ 11070 adult female from Reserva Ecológica Antisana, sector Cocodrilos, Cocodrilos-Tena road (0°39'42.50"S, 77°47'29.20"W, 1656 m), collected on 24 November 2010 by F. Velásquez-Alomoto. Provincia Pastaza: QCAZ 13585, adult male from Comunidad Zarentza, Parque Nacional Llanganates (1°21'45.47"S, 78°3'29.52"W, 1350 m), collected on 18 February 2015 by D. Rivadeneira, F. Mora, J. C. Sánchez, D. Velalcazar, D. Nuñez and J. Pinto; QCAZ 13586, adult female from Comunidad Zarentza, Parque Nacional Llanganates (1°21'45.25"S, 78°3'28.22"W, 1391 m), collected on 27 February 2015 by D. Rivadeneira, F. Mora, J. C. Sánchez, D. Velalcázar, D. Nuñez and J. Pinto.

#### Diagnosis.

**Figure 3.**
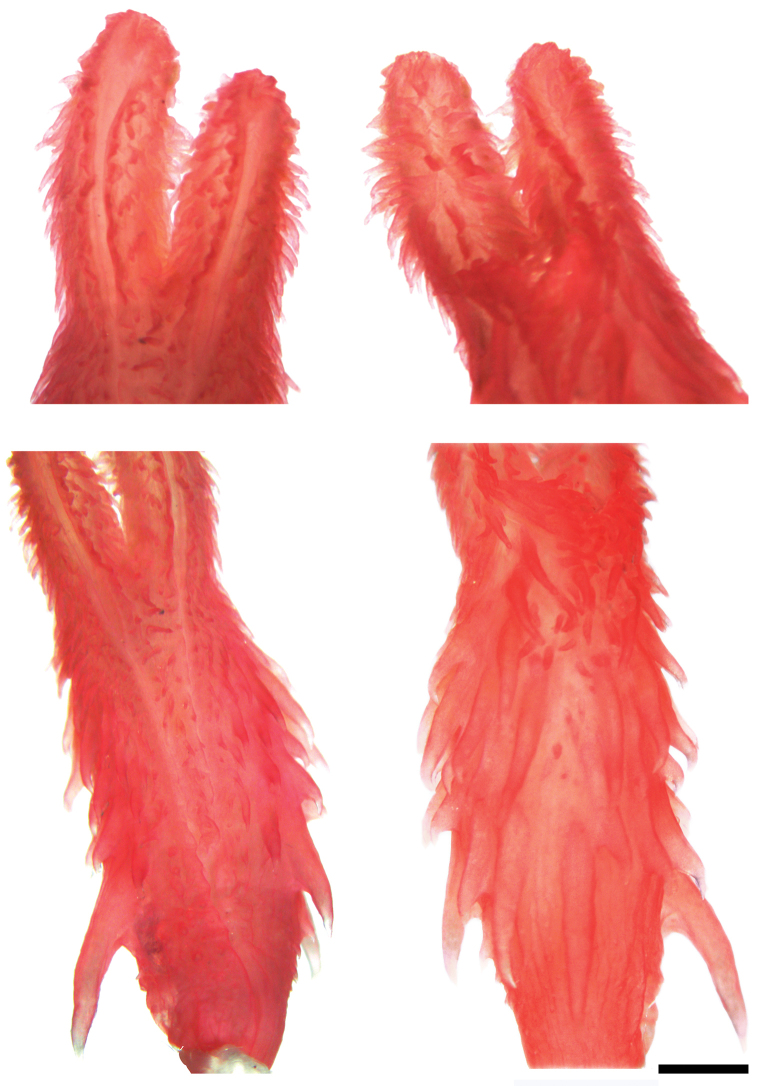
Right hemipenis of *Synophis bogerti* sp. n. (QCAZ 12791, holotype). Distal end in sulcal (upper left) and asulcal (upper right) views; body in sulcal (lower left) and asulcal (lower right) views. Scale bar = 1 mm. Photographs by Denisse Galarza.

*Synophis bogerti* can be distinguished from other species of *Synophis* by having a semicapitate, bilobed hemipenis with a large lateral spine at the base of the hemipenial body (Fig. 3); 19 longitudinal rows of dorsals at midbody; strongly keeled dorsals except for first row, which is weakly keeled (at least posteriorly); and 154–163 ventrals in males, 161–168 in females. Scutellational characters of all recognized species of *Synophis* are presented in Table 3.

**Table 3.** Summary of morphological characters and measurements (mm) of seven species of *Synophis*. Range (first line), and mean ± standard deviation (second line) are given for quantitative characters if available.

Characters	*Synophis bicolor* N = 2^1^	*Synophis bogerti* sp. n. N =7	*Synophis calamitus* N = 10^2^	*Synophis insulomontanus* sp. n. N = 4	*Synophis lasallei* N = 16^3^	*Synophis plectovertebralis* N = 2^4^	*Synophis zamora* sp. n. N = 4
Dorsal scales at midbody	19	19	19	19	21-23	19	19
Dorsal scales relief (except 1st row)	Weakly keeled	Strongly keeled	Weakly keeled	Strongly keeled	Strongly keeled	Smooth (rows 2-6) and weakly keeled	Strongly keeled
Relief of first row of dorsals	Smooth	Weakly keeled	Smooth	Keeled	Keeled	Smooth	Weakly keeled
Postoculars	2	2	1-2	2	2	-	2
Internasals	-	In contact	In contact/not in contact	In contact	In contact	In contact	In contact
Supralabials	8	8	7-8	8-9	7-9	7-8	8-9
Infralabials	9-11	10-11	8-10	10-11	10-11	7-9	9-10
Ventrals in males	184	154-163 158.25±3.77	157-165 161.4±2.97	151-152 151.5±0.71	-	144	147-153 150.75±2.63
Ventrals in females	-	161-168 164±3.6	160-166 162.88±2.23	147-149 148±1.41	-	147	-
Ventrals (sex undetermined)	180	-	-	-	144-158	-	-
Subcaudals in males	127	101-115 109.75±6.4	107-120 113±6.06	108-109 108.5±0.71	-	91	103-111 108.25±3.59
Subcaudals in females	-	98-111 105±6.56	106-113 109.67±2.42	103	-	79	-
Subcaudals (sex undetermined)	136			-	101-125	-	
Maximum total length in males (SVL)	617 (407)	641 (422)	790 (507)	541.6 (349.8)	-	212 (100)^5^	546 (359)
Maximum total length in females (SVL)	- -	603 (419)	756 (496)	467.9 (379.7)	-	272 (195.5)^5^	-

1 Data from Peracca (1896) and Nicéforo-María (1970);

2 Type specimen data from Hillis (1990);

3 Data from Hillis (1990);

4 Data from Sheil and Grant (2001);

5 Juvenile.

#### Description of the holotype.

Adult male (Figs 1, 2), SVL 367 mm; tail length 184 mm; eye diameter 1.17 mm; pupil round; head width 6.32 mm at level of supralabial 6; and head length 11.7 mm from snout to posterior margin of jaw; width at midbody 5.19 mm; head distinct from neck.

Prefrontals fused in a rectangular scale, wider than long; frontal single, with an incomplete suture from anterior margin to the middle of the scale, heptagonal, slightly wider than long; parietals large, paired, longer than wide; loreal trapezoidal, almost two times longer than high; preocular single, bordering anterior margin of orbit; supraocular single, bordering dorsal margin of orbit; temporals 1+2; anterior temporal more than two times longer than high; posterior temporals two times longer than high, approximately one half the length of anterior temporal; internasals in contact medially, distinctly wider than long; nasals not in contact; rostral visible from above, concave, nearly two times wider than long, in contact with first supralabials, nasals, and internasals; mental triangular, in contact with first pair of infralabials; infralabials 10/11; supralabials 8/8 (fourth and fifth entering orbit on both sides); anterior genials three times longer than wide, bordered laterally by infralabials 1-5 on right side, 1-6 on left side; posterior genials two times longer than wide, in contact anteromedially and separated by two gulars posteriorly, and bordered laterally by infralabials 5-6 on right side and 6-7 on left side; dorsal scale rows 19-19-17, first dorsal row weakly keeled from ventral 118, other rows strongly keeled; anal single; ventrals 163; subcaudals 115, paired.

#### Hemipenial morphology.

The following description is based on the right hemipenis of the holotype (Fig. 3; QCAZ 12791). The fully everted and maximally expanded organ is bilobed, semicalyculate, semicapitate, and extends to the sixth subcaudal. Capitular grooves are on the asulcate side; capitula are ornamented with calcified papillae, larger on the asulcate side. Numerous larger papillae meet on the asulcate side of the lobular crotch. On the sulcate side, the capitula extend along the sides of the branches of the sulcus spermaticus, far down the hemipenial body. The sulcus spermaticus bifurcates on the proximal half of the body and its branches extend centrolineally to the tip of each lobe. The hemipenial body is ornamented with large calcified spines, except on the medial region of the asulcate side, where the spines are small. The spines increase in length towards the base of the hemipenial body, with one spine on the left side (sulcate view) being considerably longer than the others. The base of the hemipenial body bears much smaller and scattered spines.

#### Color in preservative of the holotype

(Figs 1, 2). Dorsal surface of head, body and tail uniformly dark grey; skin among scales on flanks cream, visible on anterior half of body; ventrals mostly cream on anterior end of body (ventrals 1-10), becoming progressively pigmented with light grey posteriorly except on their margins; anal plate cream medially and grey laterally; subcaudals with cream margins and same tone of grey as posterior ventrals; sides of head same tone of grey as dorsal surface, except for labials, which are mostly cream ventrally; chin cream with light grey anterior margin (most of mental and first three pairs of infralabials).

#### Variation.

**Figure 4.**
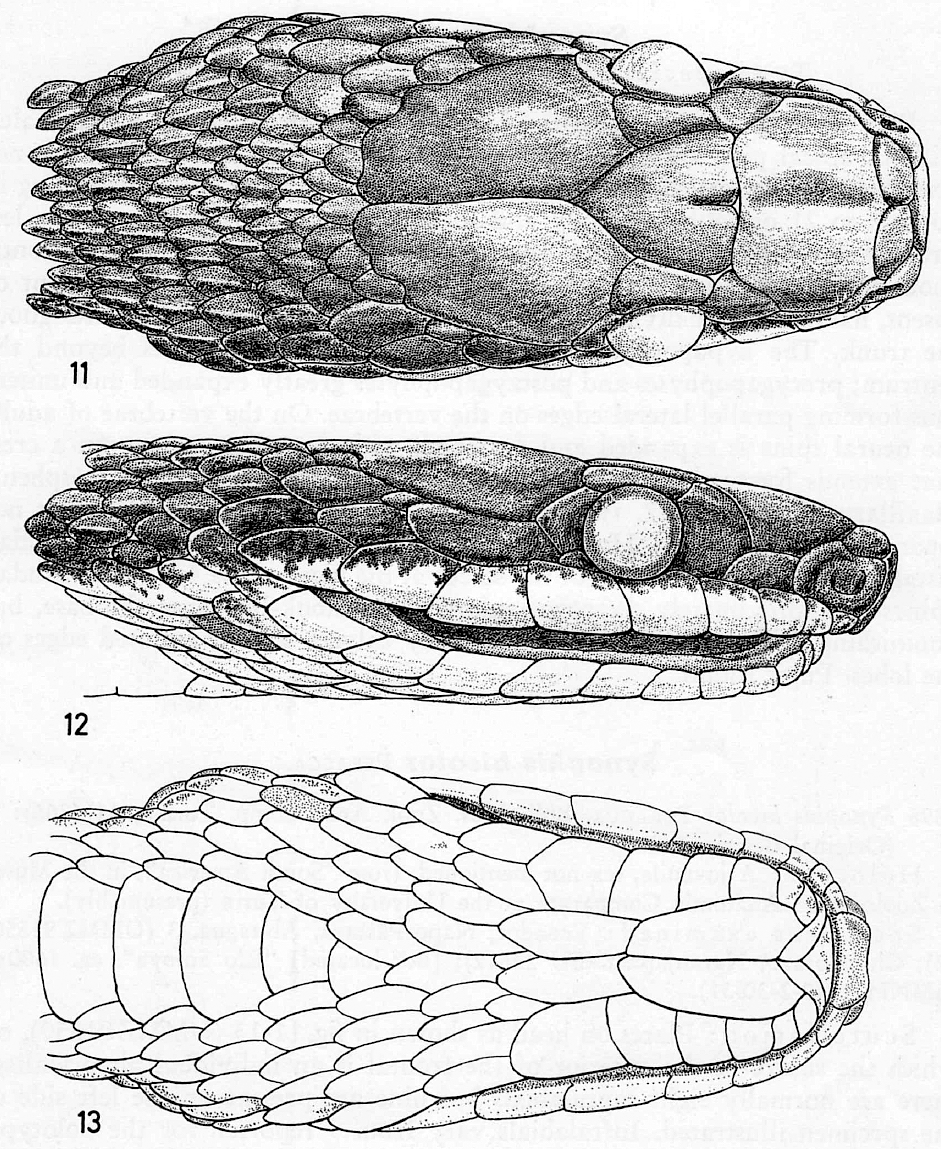
Head of specimen of *Synophis bogerti* (UMMZ 91550) illustrated by Bogert (1964) as *Synophis bicolor* showing incomplete suture on frontal scale. Illustration taken from Bogert (1964).

Intraspecific variation in scale counts and measurements in *Synophis bogerti* is presented in Table 3. Keeling on the first row of dorsals starts on ventrals 5, 10, 87, 98 and 114 in paratypes QCAZ 5072, 13323, 3511, 13585 and 11070, respectively. Besides the holotype, male paratype QCAZ 5072 is the only specimen with an incomplete medial suture on the frontal scale. This condition was also reported and illustrated by Bogert (1964) in a specimen (UMMZ 91550) from eastern Ecuador, referred by him as *Synophis bicolor* and recognized by us as *Synophis bogerti* (Fig. 4).

**Figure 5.**
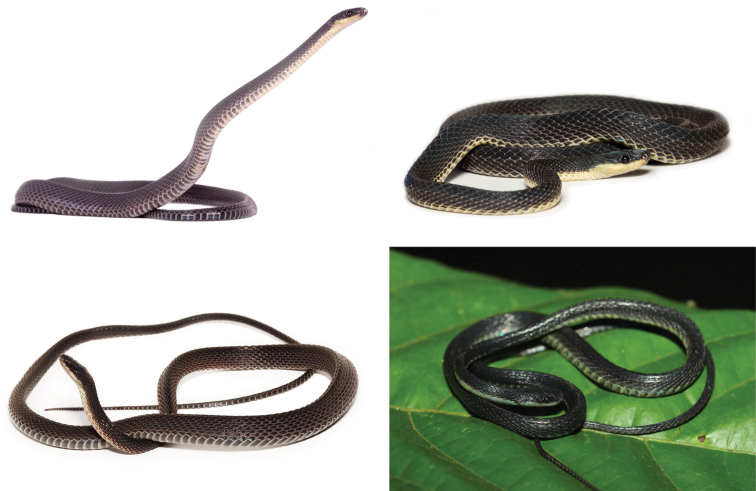
Four species of *Synophis* from Ecuador and Peru: *Synophis calamitus* (QCAZ 11931, upper left); *Synophis bogerti* sp. n. (QCAZ 13586, upper right); *Synophis zamora* sp. n. (QCAZ 13854, lower left); *Synophis insulomontanus* sp. n. (CORBIDI 13940, lower right). Photographs by Diego Quirola, Omar Torres-Carvajal and Germán Chávez.

#### Distribution and natural history.

**Figure 6.**
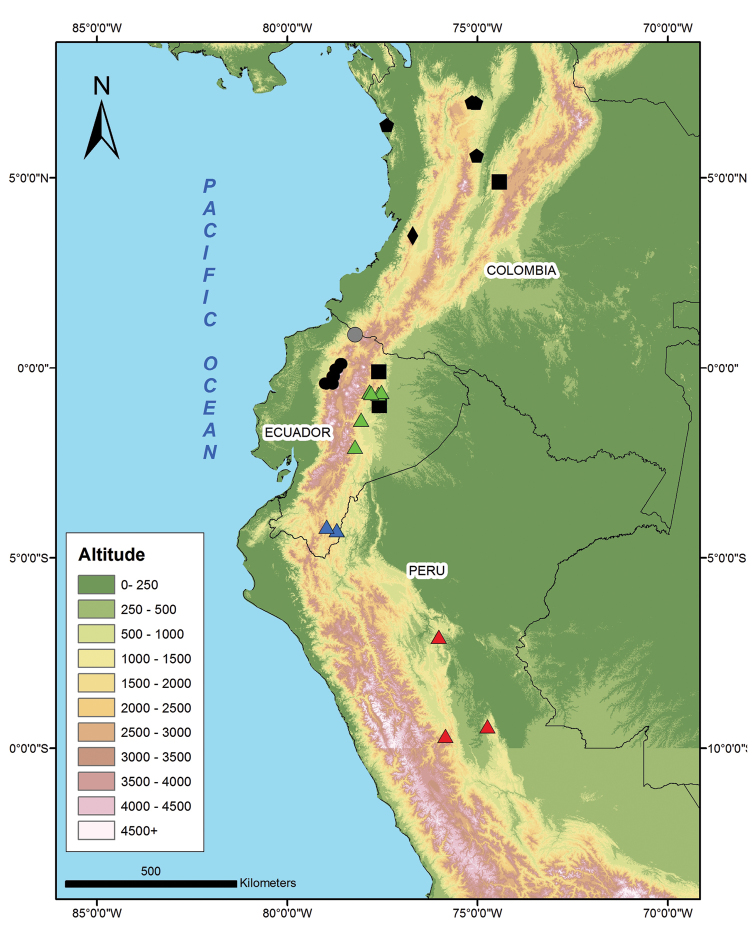
Distribution of seven species of *Synophis* in South America. *Synophis bicolor* (pentagons), *Synophis bogerti* sp. n. (green triangles), *Synophis calamitus* (circles), *Synophis insulomontanus* sp. n. (red triangles), *Synophis lasallei* (squares), *Synophis plectovertebralis* (diamond), *Synophis zamora* sp. n. (blue triangles). Grey circle corresponds to specimen QCAZ 5847 from Carchi, Ecuador (see Discussion).

*Synophis bogerti* occurs along the Amazonian slopes of the Andes in central Ecuador at elevations between 1000–1750 m (Fig. 6). The type locality is part of Wildsumaco Wildlife Sanctuary, a 400 ha reserve consisting of primary and secondary forests in a matrix of agricultural land. Most localities where *Synophis bogerti* was collected lie within protected areas including two large national parks (Llanganates and Sangay), indicating that at least some populations of this species are protected. All specimens were found active at night (20h45–00h00), mostly on the ground or on shrubs 0.5 m above ground.

#### Etymology.

The specific epithet *bogerti* is a noun in the genitive case and is a patronym for Charles M. Bogert (1908–1992), an American herpetologist and former curator of the American Museum of Natural History. Among his many contributions, Bogert published a systematic revision of *Diaphorolepis* and *Synophis*, in which he recognized that “It is also possible, of course, that specimens tentatively referred to *Synophis bicolor* are not actually conspecific” (Bogert 1964: 517). Specimens of “*Synophis bicolor*” from eastern Ecuador examined by Bogert (1964) correspond to *Synophis bogerti* sp. n.

### Synophis zamora sp. n.

Taxon classificationAnimaliaSquamataColubridae

http://zoobank.org/CAC93737-0629-4405-9E30-F1BDA841A39C

Synophis zamora Proposed standard English name: Zamoran fishing snakes
Synophis zamora Proposed standard Spanish name: Serpientes pescadoras de Zamora

#### Holotype.

**Figure 7.**
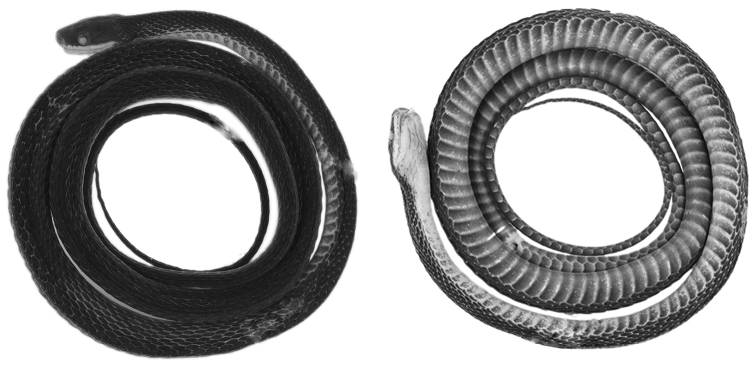
Holotype (QCAZ 9174, adult male, SVL = 349 mm) of *Synophis zamora* sp. n. in dorsal (left) and ventral (right) views. Photographs by Omar Torres-Carvajal.

**Figure 8.**
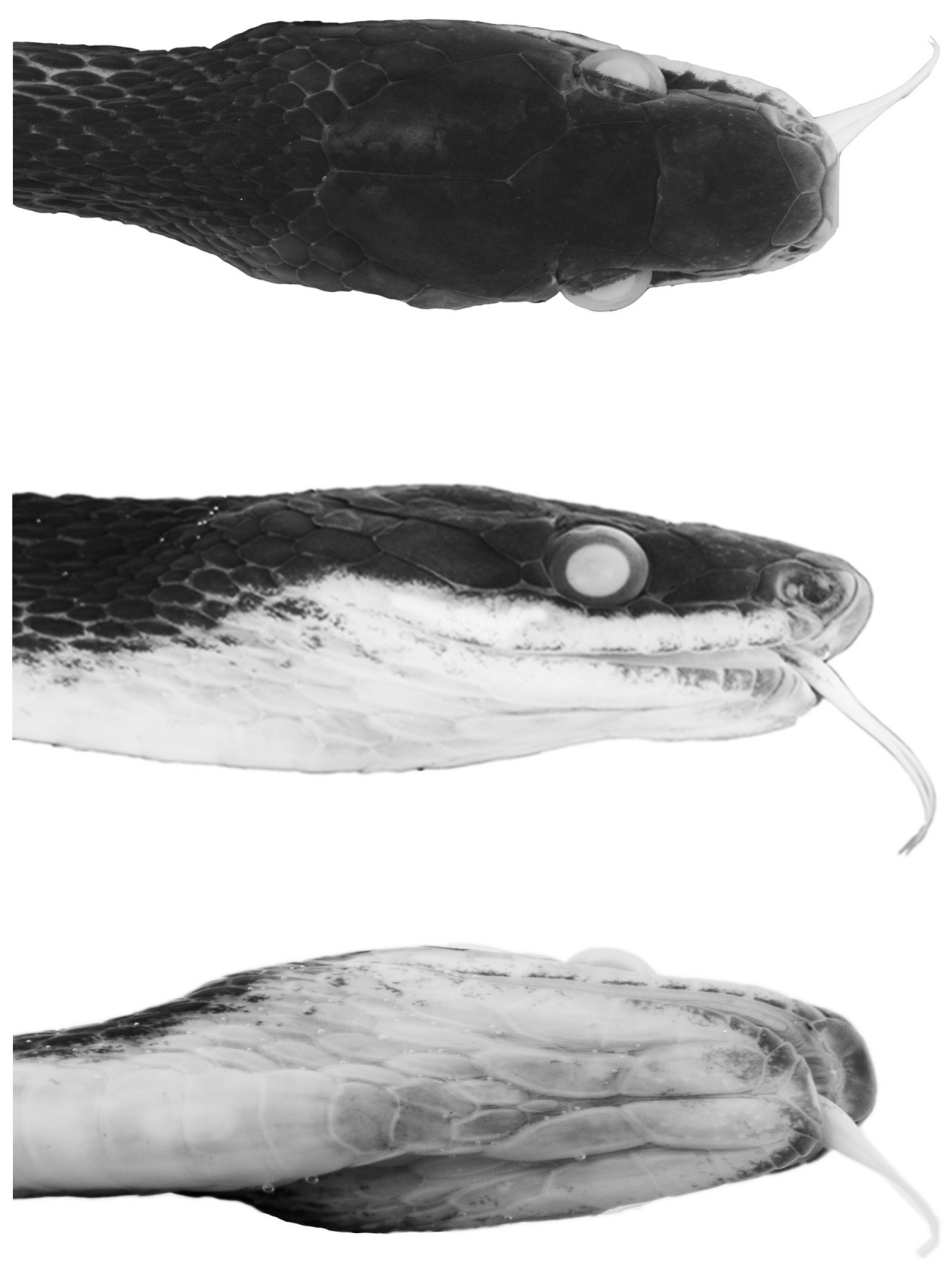
Head of holotype of *Synophis zamora* sp. n. (QCAZ 9174) in dorsal (top), lateral (middle) and ventral (bottom) views. Photographs by Omar Torres-Carvajal.

Ecuador: Provincia Zamora Chinchipe: QCAZ 9174 (Figs 7, 8), adult male from Las Orquídeas, 4 km from río Nangaritza (4°15'47.52"S, 78°41'27.93"W, 1843 m), collected on 19 April 2009 by E. E. Tapia, J. Loe Deichmann and A. F. Jiménez.

#### Paratypes.

Ecuador: Provincia Zamora Chinchipe: QCAZ 9175, adult male, same locality data as holotype; QCAZ 12773, adult male from Reserva Numbami, 18 km on road Zamora-Romerillos bajo (4°10'24.64"S, 78°57'29.63"W, 1552 m), collected on 09 July 2014 by S. R. Ron, D. A. Paucar, P.J. Venegas, D. Almeida, D. Velalcázar, M. J. Navarrete, S. Arroyo, N. Páez and Z. Lange; QCAZ 13854, adult male from Bombuscaro (4°6'42.98"S, 78°58'21.22"W, 1543 m), Podocarpus National Park, collected on 2 March 2015 by D. Rivadeneira, F. Mora, J. C. Sánchez, D. Velalcázar, D. Núñez, J. Pinto, K. Cruz and Luis T.

#### Diagnosis.

**Figure 9.**
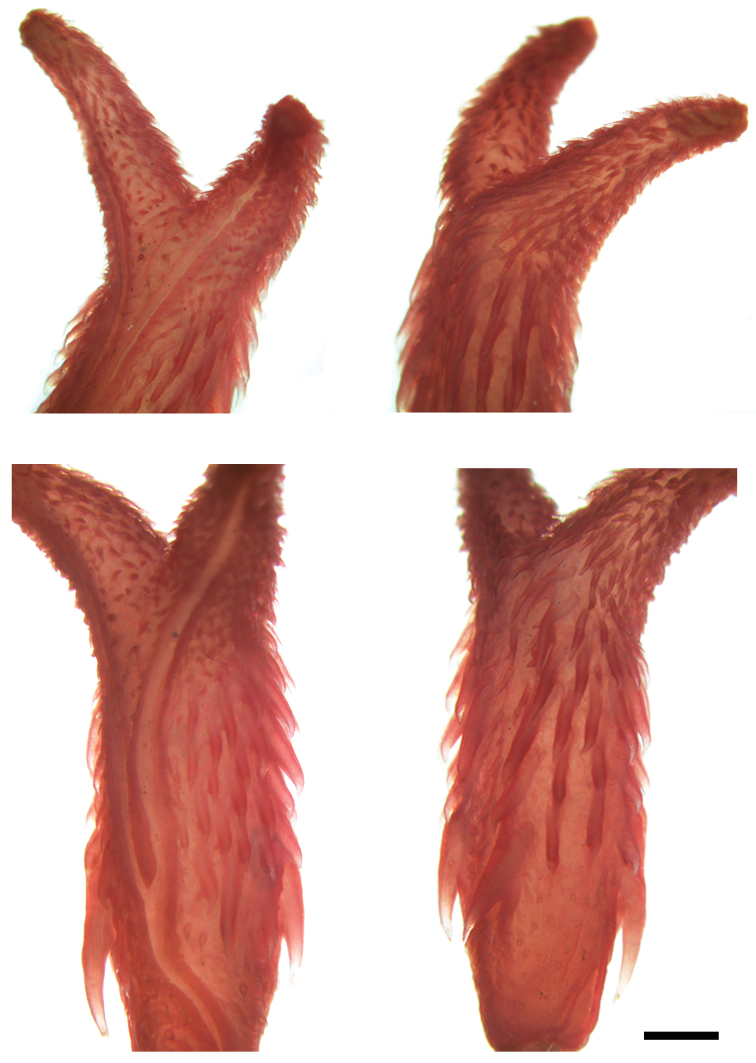
Right hemipenis of *Synophis zamora* sp. n. (QCAZ 9174, holotype). Distal end in sulcal (upper left) and asulcal (upper right) views; body in sulcal (lower left) and asulcal (lower right) views. Scale bar = 1 mm. Photographs by Denisse Galarza.

*Synophis zamora* can be distinguished from other species of *Synophis* by having a noncapitate, bilobed hemipenis with a large lateral spine at the base of the hemipenial body (Fig. 9); 19 longitudinal rows of dorsals at midbody; strongly keeled dorsals except for first row, which is weakly keeled (at least posteriorly); and 147–153 ventrals in males. Scutellational characters of all recognized species of *Synophis* are presented in Table 3.

#### Description of the holotype.

Adult male (Figs 7, 8); SVL 349 mm; tail length 185 mm; eye diameter 1.34 mm; pupil round; head width 5.15 mm at level of supralabials 6 and 7; head length 11.05 mm from snout to posterior margin of jaw; width at midbody 5.06 mm; head distinct from neck.

Prefrontals fused in a rectangular scale, wider than long; frontal single, heptagonal, slightly wider than long; parietals large, paired, longer than wide; loreal trapezoidal, two times longer than high; preocular single, bordering anterior margin of orbit; supraocular single, bordering dorsal margin of orbit; temporals 1+2; anterior temporal more than two times longer than high; posterior temporals longer than high, approximately one half the length of anterior temporal; internasals in contact medially, distinctly wider than long; nasals not in contact; rostral visible from above, concave, two times wider than long, in contact with first supralabials, nasals, and internasals; mental triangular, in contact with first pair of infralabials; infralabials 10/10; supralabials 9/9 (fourth, fifth and sixth entering orbit on both sides); anterior genials almost three times longer than wide, bordered laterally by infralabials 1-5; posterior genials three times longer than wide, in contact anteromedially and separated by three gulars posteriorly, and bordered laterally by infralabials 5-6; dorsal scale rows 19-19-17, first row weakly keeled from 15^th^ ventral, other rows strongly keeled; anal single; ventrals 147; subcaudals 103, paired.

#### Hemipenial morphology.

The following description is based on the right hemipenis of the holotype (Fig. 9; QCAZ 9174). The fully everted and maximally expanded organ is bilobed, semicalyculate, noncapitate, and extends to the sixth subcaudal. Each lobe is ornamented with small calcified papillae, slightly larger on the asulcate and lateral sides and more scattered on the sulcate side. Some larger lobular papillae meet medially at the lobular crotch on the asulcate side. The sulcus spermaticus bifurcates on the proximal half of the body and its branches extend centrolineally to the tip of each lobe. The hemipenial body is ornamented with medium-sized calcified spines, except on the medial region of the asulcate side, where the spines are small. The spines increase in length towards the base of the hemipenial body, with one spine on the left side (sulcate view) being considerably longer than the others. The base of the hemipenial body bears much smaller and scattered spines.

#### Color in preservative of the holotype

(Figs 7, 8). Dorsal surface of head, body and tail uniformly dark grey; skin among dorsal scales cream, visible on anterior half of body; ventrals cream on anterior end of body (ventrals 1-5), becoming progressively pigmented with light grey posteriorly except on their margins; anal plate cream posteriorly and grey anteriorly; subcaudals with cream margins and same tone of grey as posterior ventrals; sides of head same tone of grey as dorsal surface, except for labials, which are mostly cream; chin cream with light grey anterior margin (most of mental and first two pairs of infralabials).

#### Variation.

Intraspecific variation in scale counts and measurements in *Synophis zamora* is presented in Table 3. Keeling on the first row of dorsals starts on ventrals 9, 10, and 105 in paratypes 9175, 13854, and 12773, respectively. No major differences were found between the hemipenis of the holotype and those of paratypes QCAZ 12773 and 13854. Coloration in life (QCAZ 13854; Fig. 5) is the same as that described for the holotype above, except that the cream color has a light yellow tint.

#### Distribution and natural history.

*Synophis zamora* occurs in the southeastern portion of the northern Andes in Cordillera del Cóndor and the Amazonian slopes of the Andes at elevations between 1543–1843 m (Fig. 6). It is known from localities close to the Bombuscaro and Nangaritza rivers, which are tributaries of the Zamora river. These localities lie in Ecuador within protected areas, such as Podocarpus National Park and Numbami Ecological Reserve, indicating that at least some populations of *Synophis zamora* are protected. All specimens were found active at night (20h30-00h00), mostly on the ground or on shrubs 1-1.5 m above ground. One specimen was found on a boulder covered with moss.

#### Etymology.

The epithet *zamora* is a noun in apposition and refers to both the Zamora river and the province of Zamora Chinchipe. All type specimens were collected in this province along the upper basin of Zamora river.

### Synophis insulomontanus sp. n.

Taxon classificationAnimaliaSquamataColubridae

http://zoobank.org/0CDDA542-89E8-4DC8-B9A9-B39DF707F804

Synophis insulomontanus Proposed standard English name: Mountain fishing snakes
Synophis insulomontanus Proposed standard Spanish name: Serpientes pescadoras monteses

#### Holotype.

**Figure 10.**
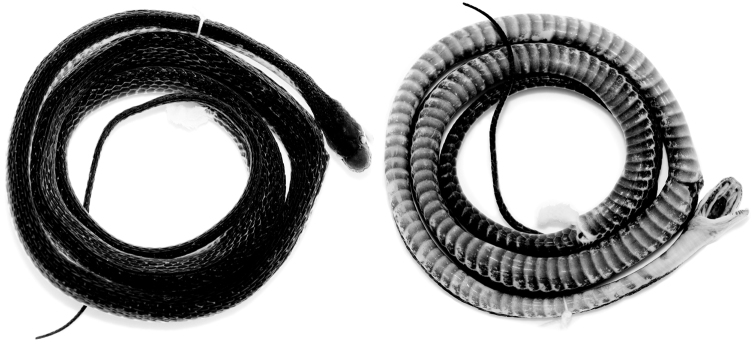
Holotype (CORBIDI 13940, adult male, SVL = 335.3 mm) of *Synophis insulomontanus* sp. n. in dorsal (left) and ventral (right) views. Photographs by Juan C. Chávez-Arribasplata.

**Figure 11.**
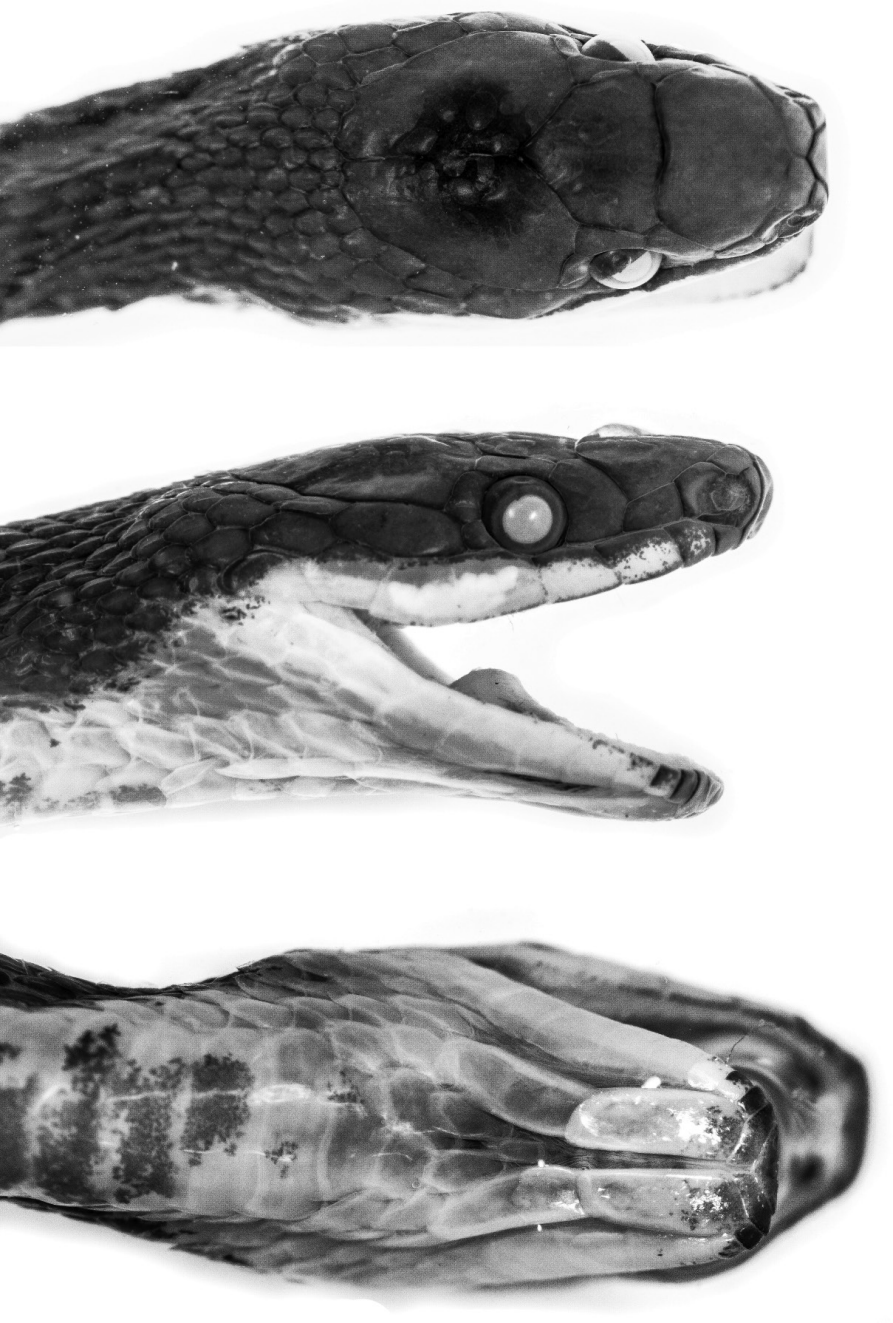
Head of holotype of *Synophis insulomontanus* sp. n. (CORBIDI 13940) in dorsal (top), lateral (middle) and ventral (bottom) views. Photographs by Germán Chávez.

Peru: Departamento Huánuco: Provincia Puerto Inca: Distrito Llullapichis: CORBIDI 13940 (Figs 10, 11), adult male from Campamento Peligroso-Reserva Comunal El Sira (9°25'34.22"S, 74°44'6.60"W, 1507 m), collected on 1 December 2013 by G. Chavez.

#### Paratypes.

Peru: Departamento San Martín: Provincia Picota: Distrito Shaboyacu: CORBIDI 9223 adult female from Parque Nacional Cordillera Azul, Puesto de Control 16 (Chambirillo) (7°4'8.90"S, 76°0'55.20"W, 1122 m), collected on 8 May 2011 by P. J. Venegas and V. Duran and CORBIDI 10418, from same locality, collected on 20 February 2012 by V. Duran. Departamento Huánuco: Provincia Huánuco: Distrito Chinchao: CORBIDI 13705 adult male from Miraflores (9°40'40.60"S, 75°50'11.09"W, 1798 m), collected 8 December 2013 by V. Duran and L. Lujan.

#### Diagnosis.

**Figure 12.**
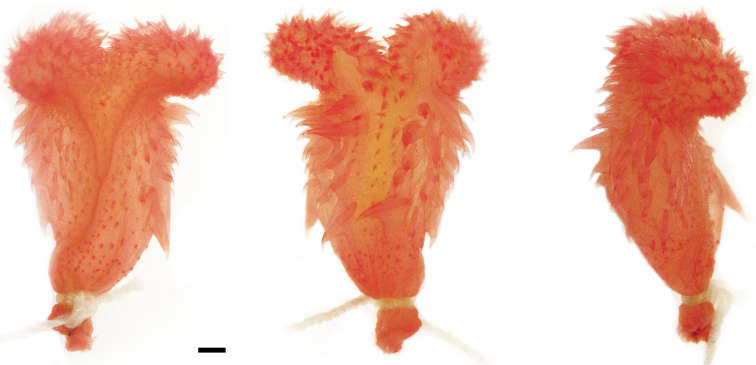
Left hemipenis of *Synophis insulomontanus* sp. n. (CORBIDI 13940, holotype) in sulcal (left), asulcal (center), and lateral (right) views. Scale bar = 1 mm. Photographs by Germán Chávez.

*Synophis insulomontanus* can be distinguished from other species of *Synophis* by having a semicapitate, bilobed hemipenis with a large lateral spine at the base of the hemipenial body, and the sulcus spermaticus bifurcating on the center of the hemipenial body (Fig. 12); 19 longitudinal rows of dorsals at midbody; strongly keeled dorsals except for first row, which is keeled to a lesser extent; 151-152 ventrals in males, 147-149 in females; 108-109 subcaudals in males, 103 in females. Scutellational characters of all recognized species of *Synophis* are presented in Table 3.

#### Description of the holotype.

Adult male (Figs 5, 10, 11), SVL 335.3 mm; tail length 180.9 mm; eye diameter 1.46 mm; pupil round; head width 6.7 mm at level of supralabial 6; head length 11.07 mm from snout to posterior margin of jaw; width at midbody 6.48 mm; head distinct from neck.

Prefrontals fused in a roughly pentagonal scale, wider than long; frontal single, pentagonal, posterior suture angular with apex directed posteriorly, wider than long; parietals large, paired, longer than wide; loreal trapezoidal, almost two times longer than high; preocular single, bordering anterior margin of orbit; supraocular single, bordering dorsal margin of orbit; temporals 1+3+3; anterior temporal more than two times longer than high; posterior temporals two times longer than high, approximately one half the length of anterior temporal; internasals in contact medially, wider than long; nasals not in contact; rostral visible from above, concave, nearly two times wider than long, in contact with first supralabials, nasals, and internasals; mental triangular, in contact with first pair of infralabials; infralabials 11/11; supralabials 8/8 (fourth and fifth entering orbit on both sides); anterior genials three times longer than wide, bordered laterally by infralabials 1-6 on both sides; posterior genials two times longer than wide, separated by gulars, and bordered laterally by infralabials 6-7 on both sides; dorsal scale rows 20-19-19, first dorsal row moderately keeled from ventral 7, other rows strongly keeled; anal single; ventrals 151; subcaudals 108, paired.

#### Hemipenial morphology.

The following description is based on the left hemipenis of the holotype (Fig. 12; CORBIDI 13940). The fully everted and maximally expanded organ is bilobed, semicalyculate, semicapitate, and extends to the fifth subcaudal. Capitular grooves are present on the asulcate side; capitula are ornamented with calcified papillae, remarkably larger on the asulcate side. A few papillae meet on the asulcate side of the lobular crotch. The sulcus spermaticus bifurcates on the center of the hemipenial body and its branches extend centrolineally to the tip of each lobe. Papillae are relatively small on the sulcate side of the hemipenial body between the bifurcating branches of the sulcus spermaticus. The hemipenial body is ornamented with large calcified spines, except on the medial region of the asulcate side and near the sulcus spermaticus, where the spines are very small. One spine on the left side (sulcate view) is considerably longer than the others. Very small spines cover the base of the hemipenial body.

#### Color in life of the holotype

(Fig. 5). Dorsal surface of head, body and tail uniformly dark grey; skin among scales on flanks cream, visible on anterior half of body; first five ventrals cream, becoming progressively pigmented with grey, except on their posterior margin where cream pigmentation is always present; anal plate grey with cream posterior border; subcaudals grey, with the porsterior borders weakly pigmented with cream in some scales; sides of head and 1^st^ supralabial same tone of grey as dorsal surface, other supralabials mostly cream; first three infralabials mostly grey, others mostly cream; scales on throat with a pale blue tone.

#### Variation.

Intraspecific variation in scale counts and measurements in *Synophis insulomontanus* is presented in Table 3. Two or three scales can be present on second row of temporals, three in the holotype and CORBIDI 9223, and two in CORBIDI 10418 and CORBIDI 13705. Paratype CORBIDI 10418 has 21 dorsals at midbody. No major differences were found between the hemipenis of the holotype and that of paratype CORBIDI 13705, except that the latter has more papillae between the bifurcating branches of the sulcus spermaticus on the sulcate side of the hemipenial body. Specimen CORBIDI 10418 has a dense cream pigmentation on ventrals from anterior end of body to midbody.

#### Distribution and natural history.

*Synophis insulomontanus* is known to occur between 1122-1798 m on the Amazonian slopes of the Andes in northern and central Peru (Fig. 6). Two localities within Departamento Huánuco, Cordillera Azul and Cordillera El Sira, correspond to sub-Andean mountain ridges, whereas the locality of Miraflores lies on the Amazonian slopes next to the Huallaga River.

The holotype was found at night, coiling inside a bromeliad, 1 m above the ground in primary premontane forest. Other specimens were found active at night, moving through leaf litter. Specimens from Cordillera Azul (CORBIDI 9223 and 10418) were found in primary premontane forest, whereas specimen CORBIDI 13705 from Miraflores, Huánuco, was found in secondary montane forest.

#### Etymology.

The epithet *insulomontanus* is a noun that derives from the Latin words *insulo* (= isolated) and *montanus* (= mountain). It refers to the isolated mountain ridges in Departamento Huánuco, where the new species was discovered.

### Phylogenetic relationships

**Figure 13.**
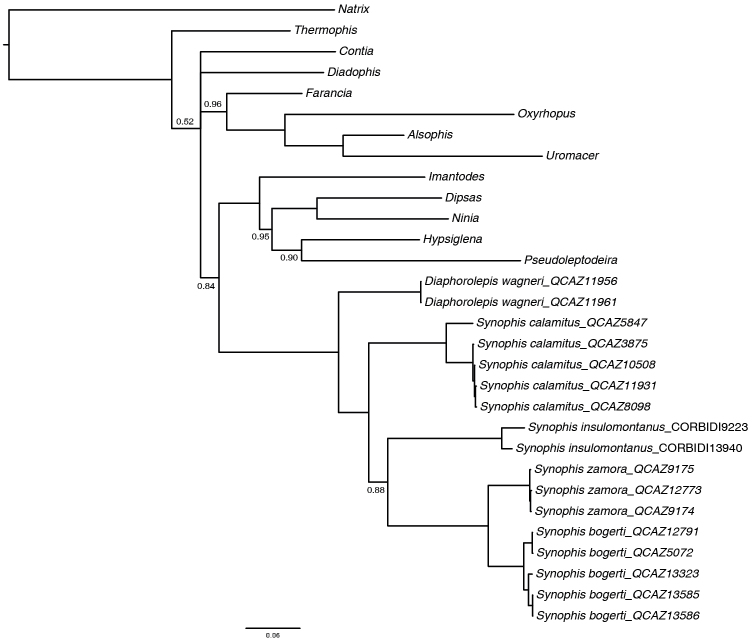
50% Majority rule consensus tree of *Synophis* snakes based on a Bayesian analysis of mtDNA sequences. Posterior probabilities are equal to 1, unless otherwise noted by numbers next to branches. Outgroup taxa are not shown.

The phylogenetic tree inferred in this study (Fig. 13) supports strongly the monophyly of *Synophis* (PP = 1). Within Dipsadinae (sensu Pyron et al. 2013), *Synophis* is sister to *Diaphorolepis* (PP = 1), and together they form a clade sister (PP = 0.84) to the strongly supported (PP = 1) clade (*Imantodes*, ((*Dipsas*, *Ninia*), (*Hypsiglena*, *Pseudoleptodeira*))).

Within *Synophis* there is a basal split into two clades, one (PP = 1) containing the trans-Andean taxon *Synophis calamitus*, and the other (PP = 0.88) including the three cis-Andean species described in this paper (*Synophis bogerti*, *Synophis insulomontanus* and *Synophis zamora*). Within the cis-Andean clade, *Synophis bogerti* and *Synophis zamora* are recovered as sister species with maximum support (PP=1), forming a clade sister to *Synophis insulomontanus*.

## Discussion

### Phylogeny of *Synophis* and *Diaphorolepis*

In spite of recent efforts to resolve the phylogenetic relationships of dipsadid snakes using DNA sequence data (e.g., Grazziotin et al. 2012; Pyron et al. 2013; Zaher et al. 2009), *Synophis* and *Diaphorolepis* have remained unsampled. Consequently, they have been considered as Dipsadidae *incertae sedis* (Zaher et al. 2009). In order to have a general idea of the phylogenetic position of both *Synophis* and *Diaphorolepis*, we included in our analysis 12 additional dipsadinae taxa used in previous phylogenetic studies (e.g., Pyron et al. 2013). We did not attempt to perform a taxonomically extensive phylogenetic analysis of Dipsadidae or Dipsadinae (sensu Pyron et al. 2013); instead, we preferred to include in our analysis only those species of Dipsadinae, for which all gene regions used in this study were available in GenBank. Our phylogeny strongly supports (1) inclusion of *Diaphorolepis* and *Synophis* within Dipsadinae; (2) a close relationship between these genera and a clade including *Imantodes*, *Dipsas*, *Ninia*, *Hypsiglena* and *Pseudoleptodeira*; and (3) a close relationship between *Diaphorolepis* and *Synophis*, as has been hypothesized using morphological evidence (Hillis 1990).

All species of *Synophis* are known to occur on Andean slopes in Colombia and Ecuador, with *Synophis insulomontanus* sp. n. representing the first record from Peru. Along with *Synophis lasallei*, the three species described in this paper are restricted to Amazonian slopes of the Andes, except for one record of *Synophis lasallei* from the western slopes of the eastern Cordillera in Colombia (Fig. 6). Thus, the Andes represent a major geographic barrier separating species of *Synophis*. Recent studies on other reptile taxa (e.g., *Alopoglossus*, *Enyalioides*) with similar distributions suggest that the uplift of the northern Andes represents a major vicariant event explaining their radiation and present distribution (Torres-Carvajal and de Queiroz 2009; Torres-Carvajal and Lobos 2014). We could not test this hypothesis with *Synophis* because we had no access to tissue samples of *Synophis bicolor*, *Synophis plectovertebralis* and *Synophis lasallei*. Nonetheless, based on morphological similarity (e.g., strongly keeled dorsals, first row of dorsals keeled; Table 3), it is likely that *Synophis lasallei* is nested in the same clade with the three eastern-Andean species described in this paper. Vertebral morphology, not examined in most species of *Synophis*, seems to support this idea. Bogert (1964) noted that the vertebrae of *Synophis* “*bicolor*” (= *Synophis bogerti* sp. n.) and *Synophis lasallei* were similar in morphology, which is different from at least *Synophis plectovertebralis* (Sheil and Grant 2001). We examined superficially trunk vertebrae of *Synophis bogerti* and *Synophis zamora* using digital X-rays, and found that the vertebrae of both species are very similar and agree with the description presented by Bogert (1964) in that “vast expansions of the prezygapophyses and postzygapophyses are coalesced as projections with relatively straight margins parallel to the main axis of each vertebra”. In addition, the zygapophyseal foramen is largely ossified as opposed to the same foramen in *Synophis plectovertebralis* (Sheil and Grant 2001). In conclusion, external and internal anatomy supports both the hypothesis presented above and the idea of a radiation of *Synophis* east of the Andes.

### Postoculars and internasals as taxonomic characters

Hillis (1990) described *Synophis calamitus* based on two specimens. Among other characters, he proposed that the number of postoculars and whether the internasals are in contact or not were useful taxonomic characters. According to Hillis (1990), *Synophis calamitus* differed from other species of *Synophis* in having one postocular (two in other species) and internasals separated by rostral and prefrontal (internasals in contact medially in other species). Among 12 specimens of *Synophis calamitus* examined in this study (Appendix I), nine have two postoculars on each side, two have one postocular on one side and two on the opposite side, and only one specimen (QCAZ 11931) has one postocular on each side. Moreover, we were able to examine the paratype of *Synophis calamitus* (KU 164208), a juvenile, badly-crushed roadkill, and found out that this specimen has one postocular on the left side and two on the right side, the ventral one difficult to observe because of the condition of the specimen. Thus, the number of postoculars is variable in *Synophis calamitus* and, therefore, it is not a useful taxonomic character. Regarding the contact between internasals, all specimens examined except for one (QCAZ 5847) had internasals in contact medially, as opposed to the condition described for both the holotype and paratype (internasals separated; Hillis 1990). Specimen QCAZ 5847 is a roadkill collected in the northern province of Carchi, and does not seem to have other differences with the remaining specimens of *Synophis calamitus* examined in this study. However, given the large branch separating this specimen from all other specimens of *Synophis calamitus* in the phylogenetic tree (Fig. 13), as well as its disjunct distribution (Fig. 6), we believe that the taxonomic status of northern (Carchi) populations should be addressed in more detail.

## Supplementary Material

XML Treatment for Synophis bogerti

XML Treatment for Synophis zamora

XML Treatment for Synophis insulomontanus

## Appendix I

**Specimens examined**

*Diaphorolepis wagneri*.—ECUADOR: **Provincia Imbabura**: QCAZ 11956, 11961, Reserva Manduriacu, 0°18'36.94"S, 78°51'27.50"W, 1213 m.

*Synophis calamitus*.—ECUADOR: **Provincia Carchi**: QCAZ 5847, Km 14 vía El Chical-Gualchán, 0°52'51.65"S, 78°13'22.80"W, 1934 m. **Provincia Cotopaxi**: QCAZ 1688, 2807, 3875, 7264, Naranjito, Bosque Integral Otonga (BIO), 0°24'53.22"S, 79°0'2.64"W, 1700 m; QCAZ 10453, Naranjito, Bosque Integral Otonga (BIO), 0°24'57.48"S, 79°0'17.28"W, 2145 m. **Provincia Pichincha**: QCAZ 381, Tandapi, 0°25'6.74"S, 78°47'58.02"W; QCAZ 1136, Chiriboga, 0°13'28.31"S, 78°46'3.90"W, 1700 m; QCAZ 3386, Cerca a Chiriboga, Las Palmeras, La Soledad, Estación Científica Río Guajalito, 0°13'44.40"S, 78°48'21.60"W, 1830 m; QCAZ 8098, 10508, Cooperativa El Porvenir, finca El Cedral, 0°6'50.40"S, 78°34'11.75"W, 2297 m; QCAZ 11931, Reserva Ecológica Bosque Nublado Santa Lucía, 0°6'56.48"S, 78°35'36.74"W, 1727 m.
